# Association Between Triglyceride-Glucose-Body Mass Index and Cardiometabolic Multimorbidity Risk in Middle-Aged and Older Chinese Adults

**DOI:** 10.3390/healthcare14213722

**Published:** 2026-11-02

**Authors:** Xiaolong Wang, Maiwulamujiang Maimaitiyiming, Lin Xia, Yuting Yang, Jialin Sun, Mingxia Jing

**Affiliations:** School of Public Health, Shihezi University, Bei’er Road, Shihezi City 832003, China

**Keywords:** triglyceride-glucose-body mass index, cardiometabolic multimorbidity, dose–response relationship, middle-aged and older adults

## Abstract

Objective: Cardiometabolic multimorbidity (CMM) typically evolves from a healthy state to an initial cardiometabolic disease (FCMD) and subsequently to CMM. The triglyceride-glucose-body mass index (TyG-BMI) is a composite surrogate marker of insulin resistance. However, its associations with incident cardiometabolic outcomes across different baseline CMD profiles remain insufficiently characterized. Using data from the China Health and Retirement Longitudinal Study (CHARLS), we examined the associations of TyG-BMI with incident first cardiometabolic disease (FCMD) among participants without CMD at baseline and incident CMM among those with one CMD at baseline. Methods: Participants were classified into two groups according to their baseline CMD status: the healthy-to-FCMD group and the FCMD-to-CMM group. Separate Cox proportional hazards models were fitted to estimate hazard ratios (HRs) and 95% confidence intervals (CIs) for each group. Dose–response relationships were evaluated using restricted cubic splines (RCS). Sensitivity analyses included a two-year landmark analysis and a complete-case analysis. Results: The healthy-to-FCMD group included 5707 participants (median follow-up: 9.0 years; 2599 incident FCMD cases), whereas the FCMD-to-CMM group included 2256 participants (median follow-up: 7.0 years; 1040 incident CMM cases). After adjustment for potential confounders, the HRs for the highest TyG-BMI quartile (Q4) compared with the lowest quartile (Q1) were 2.268 (95% CI: 2.022–2.544) for FCMD and 1.895 (95% CI: 1.574–2.282) for CMM. Each 1-standard deviation (SD) increase in TyG-BMI was associated with a 38.5% higher hazard of FCMD (HR = 1.385, 95% CI: 1.332–1.440) and a 31.5% higher hazard of CMM (HR = 1.315, 95% CI: 1.232–1.404). RCS analyses indicated positive associations between TyG-BMI and both outcomes, with no statistically significant evidence of nonlinearity within the observed range (*p* for nonlinearity = 0.963 and 0.150, respectively). The associations were generally consistent in sensitivity analyses. Conclusion: Higher TyG-BMI was associated with an increased hazard of incident FCMD among participants without CMD at baseline and incident CMM among those with one CMD at baseline. These findings provide complementary observational evidence on cardiometabolic risk across different baseline CMD profiles.

## 1. Introduction

Cardiometabolic diseases (CMDs) remain leading causes of death worldwide. These conditions frequently coexist within individuals, a condition referred to as cardiometabolic multimorbidity (CMM). CMM is defined as the coexistence of two or more cardiometabolic diseases (CMDs), such as hypertension, diabetes, stroke, and heart disease [1]. Among different patterns of multimorbidity, CMM is particularly concerning because it is associated with approximately twice the mortality risk observed in individuals with a single CMD or other forms of multimorbidity [2,3,4]. Consequently, CMM represents a substantial public health challenge [5].

The progression of CMM typically unfolds sequentially: beginning with a healthy state, transitioning to first cardiometabolic disease (FCMD), and ultimately advancing to CMM [6]. Identifying modifiable risk factors associated with these stages is important for prevention. A recent systematic review and meta-analysis identified stage-specific risk factors, highlighting overweight and obesity as important modifiable factors associated with both the initial development of CMD and the subsequent occurrence of CMM [7]. Insulin resistance (IR) is a key mechanism linking obesity to cardiometabolic diseases and involves visceral fat accumulation and dysregulation of glucose and lipid metabolism [8]. Therefore, a composite indicator incorporating glucose metabolism, lipid metabolism, and adiposity may be useful for assessing cardiometabolic risk.

The triglyceride-glucose-body mass index (TyG-BMI) combines the triglyceride-glucose (TyG) index with body mass index (BMI), providing a practical surrogate measure of IR that incorporates these three dimensions. This index is simple, cost-effective, and suitable for repeated measurement during follow-up. Higher TyG-BMI levels have been associated with increased risks of hypertension, diabetes, coronary heart disease, and stroke [9,10,11]. However, most previous studies have focused on individual disease outcomes or overall multimorbidity accumulation. Evidence regarding the associations between TyG-BMI and incident cardiometabolic outcomes across different baseline CMD profiles remains limited, particularly among middle-aged and older Chinese adults.

To address this gap, we used data from the China Health and Retirement Longitudinal Study (CHARLS) to examine the associations of TyG-BMI with incident cardiometabolic outcomes in two separate analytical groups defined by baseline CMD status. Specifically, we investigated incident FCMD among participants without CMD at baseline and incident CMM among those with exactly one CMD at baseline. Separate Cox proportional hazards models and restricted cubic spline analyses were conducted for each group to assess these associations and potential nonlinear dose–response relationships. This analytical approach was intended to provide complementary evidence on cardiometabolic risk across different levels of baseline CMD burden rather than to model sequential within-person transitions between disease states.

## 2. Materials and Methods

### 2.1. Study Population

The China Health and Retirement Longitudinal Study (CHARLS) is a nationally representative, ongoing prospective cohort study focusing on Chinese adults aged 45 and older. Initiated in 2011–2012, the baseline survey employed a multistage stratified probability sampling design, successfully enrolling 17,708 participants from 150 counties/districts and 450 communities/villages across 28 provinces. Follow-up assessments occur every 2 to 3 years. The study protocol received approval from the Biomedical Ethics Review Committee of Peking University (approval number: IRB00001052-11015), and all participants provided written informed consent [12].

We analyzed data from five waves of CHARLS (2011–2020). Participants were excluded based on the following criteria: (1) Age below 45 years or missing age data (*n* = 508). (2) Missing data required for TyG-BMI calculation (*n* = 7934). Specifically, missing information involved fasting plasma glucose (FPG, *n* = 6072), triglycerides (TG, *n* = 6052), and body mass index (BMI, *n* = 4077), and participants were excluded if any of these components was unavailable. (3) Missing information on any of the four CMDs (*n* = 445). (4) Having CMM at baseline (*n* = 653). (5) Having extreme TyG-BMI values (*n* = 205). Ultimately, 7963 participants were included in the final analysis.

**Figure 1:**
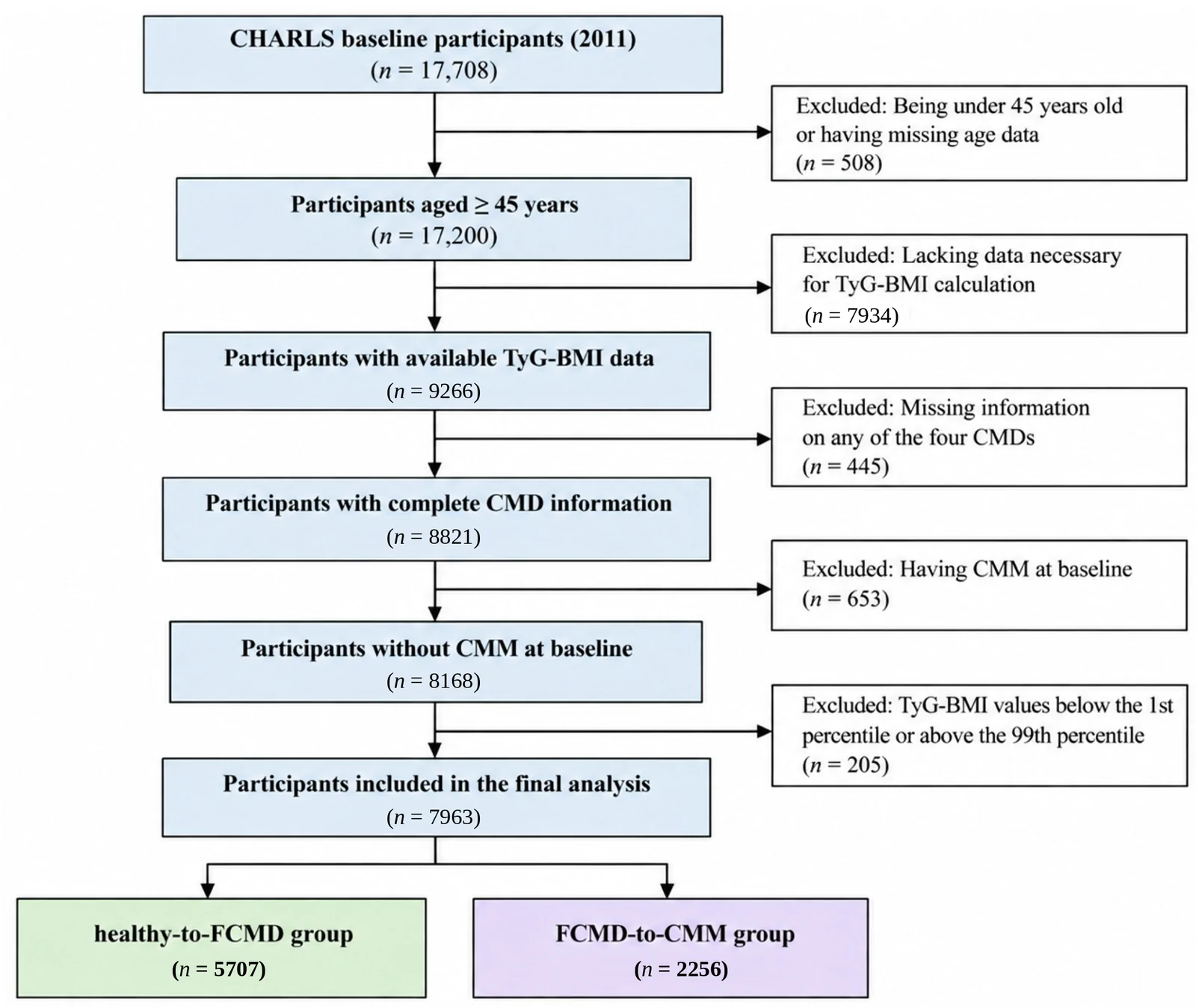
Flowchart for Inclusion/Exclusion of Study Participants.

Participants were classified into two separate analytical groups according to their baseline CMD status. The healthy-to-FCMD group included individuals without any CMD at baseline (*n* = 5707), whereas the FCMD-to-CMM group comprised participants with exactly one CMD at baseline (*n* = 2256). Separate Cox proportional hazards models were used to examine incident FCMD and incident CMM, respectively. These analyses did not constitute a multistate model of successive disease-state transitions within the same individuals. The detailed participant selection process is illustrated in Figure 1.

### 2.2. TyG-BMI

TyG-BMI is a composite surrogate marker of insulin resistance (IR) that incorporates glucose metabolism, lipid metabolism, and adiposity. Fasting venous blood samples were collected after an overnight fast of at least 8 h. TyG-BMI was calculated among participants with available fasting blood biomarker data. Fasting plasma glucose (FPG) and triglyceride (TG) concentrations were measured using enzymatic colorimetric assays based on the blood biomarker dataset from the China Health and Retirement Longitudinal Study (CHARLS) [13]. Both FPG and TG were recorded in mg/dL and used without additional unit conversion. BMI was calculated as body weight in kilograms divided by height in meters squared (kg/m^2^). Height recorded in centimeters in CHARLS was converted to meters before BMI calculation. The TyG index was calculated using the following formula:

$$\mathrm{TyG}\;=\;\ln(\frac{\mathrm{FPG}\times \mathrm{TG}}{2})$$

where ln denotes the natural logarithm.

The TyG-BMI was calculated as:

*TyG*-BMI = *TyG* × *BMI*

### 2.3. Ascertainment of Cardiometabolic Diseases

Four cardiometabolic diseases (CMDs) were considered in this study: hypertension, diabetes, heart disease, and stroke. CMD status was determined from self-reported physician diagnoses in the CHARLS health-status questionnaires. Specifically, a participant was classified as having a CMD if they reported having been diagnosed with the corresponding condition by a physician. This self-reported physician-diagnosis-based definition was applied consistently across the 2011, 2013, 2015, 2018, and 2020 survey waves. Although objective measurements (blood pressure, blood glucose, HbA1c) and medication-related data were available for selected survey waves, these metrics were not incorporated into our CMD outcome definitions.

Participants were classified into two groups according to their baseline CMD status. The healthy-to-FCMD group included participants who had none of the four CMDs at baseline, and the outcome was defined as the first observed occurrence of any of these CMDs during follow-up. The FCMD-to-CMM group included participants who had exactly one CMD at baseline, and the outcome was defined as the first observed presence of two or more CMDs during follow-up.

During follow-up, CMD status was assessed using available disease information from successive CHARLS waves. Participants who missed intermediate interviews were retained if data from subsequent waves were available. Disease status was assessed independently at each observed wave; previous diagnoses were not automatically carried forward, and missing disease information was not treated as evidence of disease absence. For the healthy-to-FCMD group, the event time was defined as the first follow-up assessment identifying any of the four CMDs; for the FCMD-to-CMM group, it was the first assessment identifying two or more CMDs at the same follow-up assessment. These event times reflect the first observed detection rather than the exact time of disease onset.

Follow-up accrued from the 2011 baseline until the first qualifying event, death, or the last available interview, whichever occurred first. Participants who died before experiencing the outcome were censored at the time of death in the primary Cox analyses. Competing-risk regression was not performed.

### 2.4. Covariates

The multivariable models incorporated the following covariates: age (continuous, measured in years), sex (male or female), marital status (married or unmarried), residence (urban or rural), education level (categorized as less than primary school, primary school, middle school, or high school and above), total annual household income (continuous, in yuan), drinking status (ever or currently drinking), smoking status (ever or currently smoking), and sleep duration (continuous, in hours).

Covariates were selected a priori based on previous literature and clinical plausibility, considering their potential associations with cardiometabolic disease risk and their potential roles as confounders. As TyG-BMI is mathematically derived from fasting plasma glucose, triglycerides, and BMI, these individual components were not additionally included as covariates in the multivariable models to avoid potential multicollinearity and difficulties in interpreting the association of the composite index with cardiometabolic outcomes.

### 2.5. Statistical Analysis

To manage extreme values, participants with TyG-BMI values outside the 1st–99th percentile range were excluded (percentile-based trimming). Missing covariate data were handled using multiple imputation by chained equations (MICE). The imputation procedure was performed separately for the healthy-to-FCMD group and the FCMD-to-CMM group. The imputation models included all covariates used in the multivariable Cox proportional hazards models, including age, sex, marital status, residence, education level, total annual household income, drinking status, smoking status, and sleep duration. TyG-BMI, follow-up time, and event status were incorporated as auxiliary variables in each imputation model. Participants with missing TyG-BMI values were excluded prior to imputation; thus, TyG-BMI itself was not imputed. Five imputed datasets were generated with 20 iterations under the missing-at-random (MAR) assumption. Cox proportional hazards models were fitted within each imputed dataset, and pooled effect estimates were obtained using Rubin’s rules. Continuous variables are presented as either mean ± standard deviation (SD) or median (Q1, Q3), while categorical variables are shown as frequencies and percentages (%).

Cox proportional hazards models were fitted to estimate hazard ratios (HRs) and 95% confidence intervals (CIs). Three models were specified: an unadjusted model, an age- and sex-adjusted model, and a fully adjusted model. Cause-specific Cox proportional hazards models were used for the primary analyses, with death before the occurrence of the outcome treated as a censoring event. TyG-BMI was categorized into quartiles, with Q1 as the reference, and trend tests were performed.

RCS Cox models were fitted to each of the five imputed datasets. Regression coefficients and covariance matrices were pooled using Rubin’s rules, and multivariable Wald chi-square tests based on the pooled estimates were used to evaluate overall and nonlinear associations. The proportional hazards assumption was evaluated using Schoenfeld residuals (cox.zph function in R) for the fully adjusted Cox models across all five imputed datasets in each group. No statistically significant violations were detected for TyG-BMI or in the global tests, although household income showed evidence of a potential violation in some imputed datasets in the healthy-to-FCMD group.

Sensitivity analyses included a two-year landmark analysis and a complete-case analysis. In the two-year landmark analysis, participants who did not remain under observation beyond two years after baseline were excluded, and follow-up time was recalculated from the two-year landmark. Fully adjusted Cox proportional hazards models were then refitted.

Additional subgroup analyses were conducted in the FCMD-to-CMM group according to the type of CMD present at baseline (hypertension, diabetes, heart disease, or stroke). Multivariable Cox proportional hazards models were fitted separately for each subgroup to examine the association between TyG-BMI and the risk of incident CMM. Potential effect modification by baseline CMD type was assessed by including an interaction term between TyG-BMI and baseline CMD type in the fully adjusted Cox model. The overall interaction was evaluated using the D1 test across five multiply imputed datasets. Exploratory interaction analyses further compared each baseline CMD category with the other three categories combined, with Holm adjustment for multiple comparisons.

Missingness for each covariate before multiple imputation is provided in Supplementary Table S1. Restricted cubic spline knot positions for TyG-BMI are reported in Supplementary Table S2. The complex CHARLS sampling design, including sampling weights, stratification, and clustering, was not incorporated into the analyses. All analyses were performed using R 4.4.1. All statistical tests were two-sided, and *p* < 0.05 was defined as the threshold for statistical significance.

## 3. Results

### 3.1. Study Population and Baseline Characteristics

The healthy-to-FCMD group had a mean age of 58.3 ± 9.2 years, of whom 53.5% were women and 67.3% lived in rural areas. The mean TyG-BMI was 196.7 ± 33.8. In contrast, the FCMD-to-CMM group was slightly older, averaging 61.2 ± 9.1 years, with a higher percentage of females at 59.6% and a higher TyG-BMI of 212.6 ± 36.9. The two groups showed similarities with respect to marital status, residence, educational attainment, household income, and lifestyle-related factors. Given the observational study design, formal between-group hypothesis tests of baseline characteristics were not performed (Table 1). Within the FCMD-to-CMM group (*n* = 2256), hypertension was the most common baseline CMD (n = 1513, 67.07%), followed by heart disease (*n* = 464, 20.57%), diabetes (*n* = 211, 9.35%), and stroke (*n* = 68, 3.01%). The distribution of baseline CMDs and the corresponding numbers of incident CMM cases are presented in Supplementary Table S3.

### 3.2. Association of TyG-BMI with FCMD and CMM Risk

**Figure 2:**
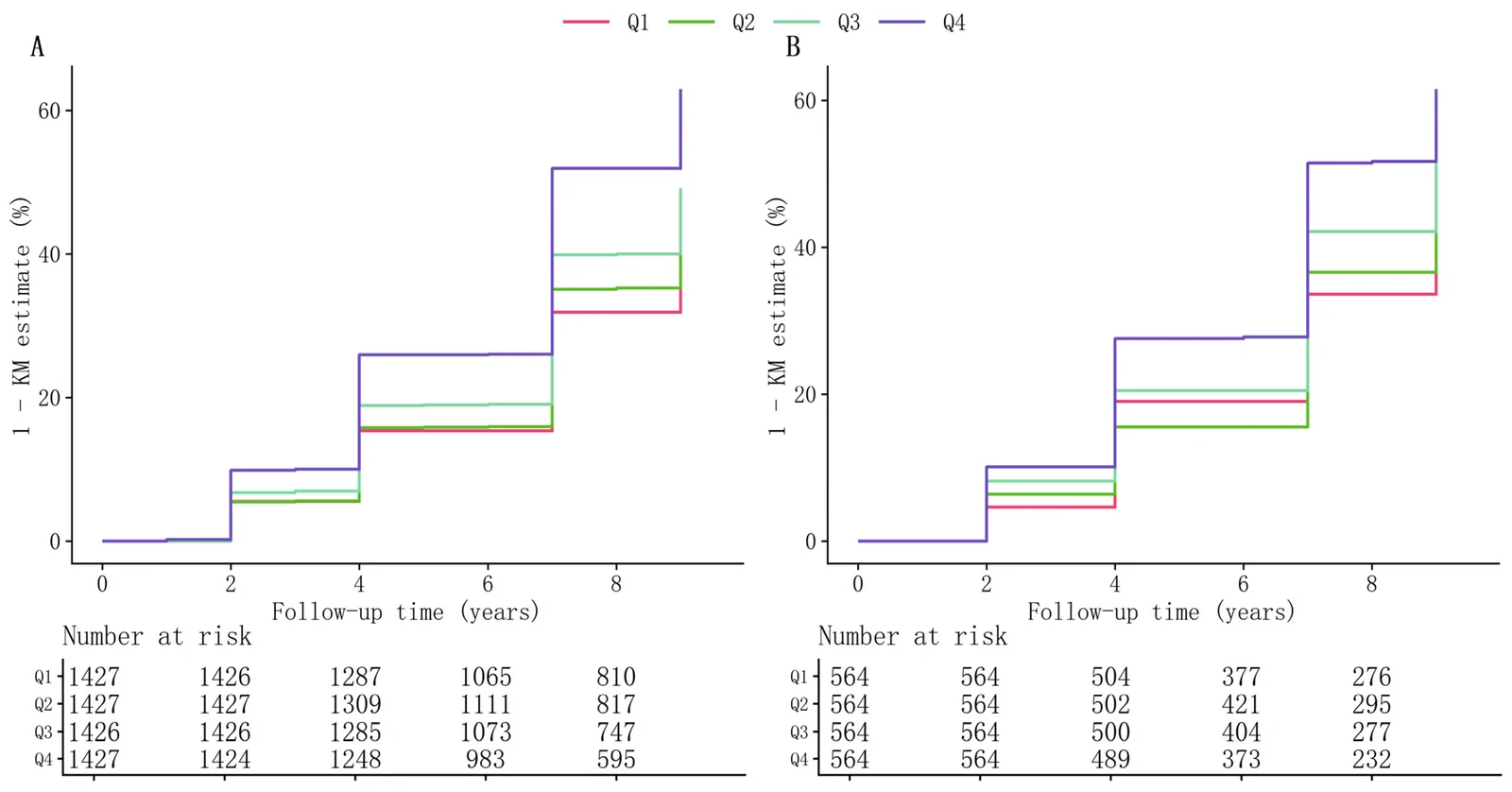
Kaplan–Meier-based estimates of event occurrence by TyG-BMI quartiles in the two groups. Note: (**A**) healthy-to-FCMD group (*n* = 5707), outcome FCMD; (**B**) FCMD-to-CMM group (*n* = 2256), outcome CMM. The curves represent one minus the Kaplan–Meier survival estimate. Deaths before the outcome were treated as censoring events. Accordingly, these estimates do not account for competing mortality and should not be interpreted as cumulative incidence probabilities accounting for competing risks.

During follow-up, the healthy-to-FCMD group had a median follow-up duration of 9.0 years (range: 1.0–9.0 years), with 2599 incident FCMD cases and an incidence rate of 64.49 per 1000 person-years. In contrast, the FCMD-to-CMM group had a median follow-up of 7.0 years (range: 2.0–9.0 years), with 1040 incident CMM cases and an incidence density of 67.61 per 1000 person-years. Kaplan–Meier-based estimates of event occurrence were higher in the higher TyG-BMI quartiles in both analytical groups (Figure 2).

In the fully adjusted model (Model 3), the hazard ratio (HR) for FCMD comparing the highest TyG-BMI quartile (Q4) with the lowest quartile (Q1) in the healthy-to-FCMD group was 2.268 (95% CI: 2.022–2.544). Similarly, in the FCMD-to-CMM group, the *HR* for CMM in the Q4 group was 1.895 (95% *CI*: 1.574–2.282). The *p* values for trend were <0.001 in both groups, indicating statistically significant trends across TyG-BMI quartiles (Table 2).

In the FCMD-to-CMM group, additional analyses stratified by baseline CMD type showed that higher TyG-BMI was significantly associated with an increased hazard of incident CMM among participants with baseline hypertension (HR = 1.0065, 95% CI: 1.0043–1.0088), heart disease (HR = 1.0118, 95% CI: 1.0078–1.0158), and stroke (HR = 1.0268, 95% CI: 1.0105–1.0434). The association was not statistically significant among participants with baseline diabetes (HR = 1.0050, 95% CI: 0.9992–1.0109; *p* = 0.091). The overall interaction between TyG-BMI and baseline CMD type was statistically significant (*p* for interaction = 0.0334). However, exploratory interaction tests comparing each baseline CMD category with the remaining three categories combined were not statistically significant (all *p* for interaction >0.05). Detailed results are provided in Supplementary Table S3.

### 3.3. Dose–Response Relationship Between TyG-BMI and FCMD and CMM Risk

**Figure 3:**
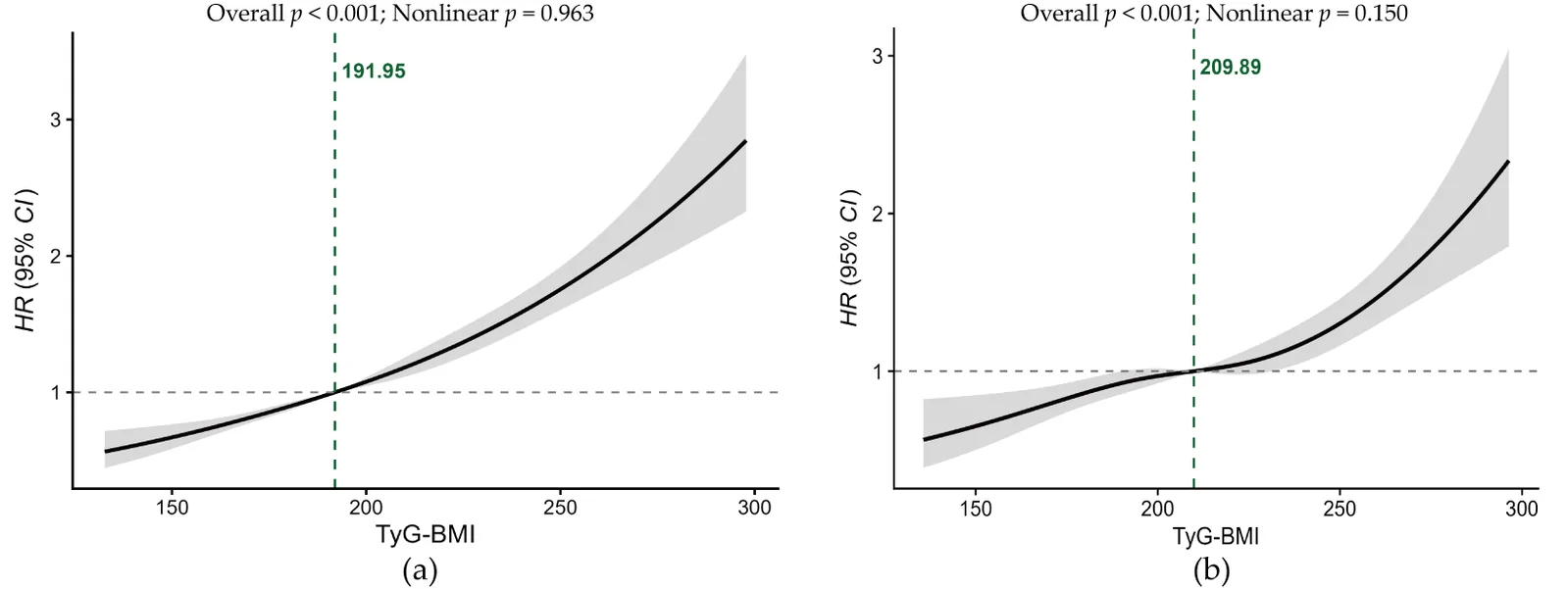
Restricted cubic spline analysis of the association between TyG-BMI and FCMD and CMM risk. Note: (**a**) healthy-to-FCMD group, outcome FCMD; (**b**) FCMD-to-CMM group, outcome CMM. The solid black line represents hazard ratios (HRs), and the grey shaded area indicates the 95% confidence interval (CI). The horizontal grey dashed line denotes HR = 1. The vertical dark-green dashed line indicates the median value of TyG-BMI, which was set as the reference point (HR = 1). Restricted cubic spline Cox models were fully adjusted for age, sex, marital status, residential location, educational attainment, total annual household income, drinking status, smoking status, and sleep duration. Four knots were specified for TyG-BMI, with knot positions determined separately for each analytical group. The displayed RCS curves and 95% confidence intervals were obtained from the first imputed dataset, whereas the overall and nonlinearity *p* values were calculated from pooled Wald tests across all five imputed datasets.

RCS analyses were performed to assess dose–response associations between TyG-BMI and incident FCMD and CMM in the respective analytical groups. In both groups, the RCS curves suggested positive associations between TyG-BMI and the outcomes. However, the tests for nonlinearity did not provide statistically significant evidence of nonlinear associations (*p* for nonlinearity = 0.963 for the healthy-to-FCMD group and 0.150 for the FCMD-to-CMM group) (Figure 3).

### 3.4. Sensitivity Analysis

In the two-year landmark analysis, the associations between TyG-BMI and FCMD risk remained consistent with the main analysis in the healthy-to-FCMD group (HR: 1.009, 95% CI: 1.008–1.011, *p* < 0.001). Similarly, the association between TyG-BMI and incident CMM remained stable in the FCMD-to-CMM group (HR: 1.007, 95% CI: 1.005–1.009, *p* < 0.001). Comparable results were observed in the complete-case analysis (Table 3).

## 4. Discussion

In this study, we used CHARLS data to examine two groups defined by baseline CMD status: the healthy-to-FCMD group and the FCMD-to-CMM group. We found that higher TyG-BMI was associated with a higher hazard of incident FCMD among participants without CMD at baseline and incident CMM among those with one CMD at baseline, with no statistically significant evidence of nonlinear associations in either group. After adjustment for potential confounders, each 1-standard deviation (SD) increase in TyG-BMI was associated with a 38.5% higher hazard of FCMD among participants without CMD at baseline and a 31.5% higher hazard of CMM among those with one CMD at baseline.

In the FCMD-to-CMM group, subgroup analyses suggested potential heterogeneity in the association between TyG-BMI and incident CMM across baseline CMD types. Although the overall interaction test was statistically significant, exploratory interaction tests comparing individual baseline CMD categories with the remaining categories were not statistically significant. These findings should be interpreted cautiously because of the multiple exploratory comparisons and small sample sizes in certain subgroups, particularly the stroke subgroup.

Our results are broadly consistent with previous studies reporting positive associations between TyG-BMI and cardiometabolic disease outcomes [14,15,16,17,18]. Notably, Han et al. [14] used a multistate model based on UK Biobank data to investigate the associations of insulin resistance-related indices, including TyG-BMI, with successive cardiometabolic disease states. In contrast, we conducted separate Cox proportional hazards analyses in two groups defined by baseline CMD status, examining incident FCMD among participants without CMD and incident CMM among those with one CMD at baseline. Thus, rather than estimating successive disease-state transitions within a unified multistate framework, our analysis provides complementary evidence on the association between TyG-BMI and cardiometabolic outcomes in middle-aged and older Chinese adults with different baseline disease burdens. The positive associations observed in both groups were consistent in direction with previous findings, although differences in populations, outcome definitions, and analytical approaches should be considered when comparing effect estimates.

The shape of the association observed in our study differs from that reported in some previous studies. Research based on CHARLS and the Atherosclerosis Risk in Communities (ARIC) study has reported significant nonlinear relationships between TyG-BMI and CMM [19,20]. Previous research has also suggested that insulin resistance-related indices exhibit stage-specific associations in the development of CMM [21]. These discrepancies may reflect differences in baseline CMD profiles, outcome definitions, and analytical methods. Whereas other studies assessed overall CMM incidence or modelled sequential disease-state transitions, the present analysis evaluated two distinct incident endpoints within subgroups defined by baseline cardiometabolic burden. These explanations remain speculative and warrant further investigation.

Despite differences in dose–response patterns across studies, the observed associations of TyG-BMI with incident FCMD and CMM are biologically plausible. TyG-BMI integrates glucose metabolism, lipid metabolism, and adiposity, and its associations with incident cardiometabolic outcomes may reflect the combined effects of metabolic disturbances. In obesity, excess subcutaneous fat storage can lead to adipocyte hypertrophy and chronic low-grade inflammation, increasing circulating free fatty acids and promoting visceral and ectopic fat deposition. These processes may contribute to a self-reinforcing cycle of insulin resistance (IR) and inflammation [22]. Compensatory hyperinsulinemia associated with IR may impair endothelial nitric oxide synthase activation through inhibition of the PI3K/Akt pathway, reducing nitric oxide bioavailability and increasing endothelin-1 expression, thereby promoting vasoconstriction and atherosclerosis [23]. Concurrently, IR-related lipid abnormalities, including reduced high-density lipoprotein levels, elevated low-density lipoprotein levels, and increased triglyceride concentrations, may further promote lipid deposition. Excess free fatty acids may also activate the MAPK pathway, increasing free radical formation and exacerbating IR [24]. These interconnected pathways may contribute to the development of cardiometabolic diseases and multimorbidity. As TyG-BMI is a composite index incorporating adiposity, glucose metabolism, and lipid metabolism, its observed associations with cardiometabolic outcomes should be interpreted as reflecting aggregate metabolic burden rather than independent causal effects of its individual components.

Although TyG-BMI can be calculated from routinely measured clinical parameters, the observed associations do not establish its utility for clinical screening, monitoring, or risk prediction. Further studies evaluating discrimination, calibration, incremental predictive value beyond established risk factors, and clinical utility are needed before TyG-BMI can be recommended for these purposes. Moreover, whether interventions targeting TyG-BMI or its components can improve cardiometabolic outcomes remains uncertain.

This study has several limitations. First, its observational design precludes causal inference, and residual confounding from unmeasured factors, such as physical activity and diet, may remain. Second, CMD outcomes were based on self-reported physician diagnoses, and inconsistent reports across survey waves may have introduced misclassification. Intermittent follow-up assessments and missing intermediate interviews may also have introduced uncertainty in event timing, as exact disease onset dates were unavailable. Third, death before FCMD or CMM was treated as a censoring event in the cause-specific Cox models, without competing-risk analysis; thus, the estimated hazard ratios should not be interpreted as cumulative incidence estimates accounting for competing mortality. Fourth, excluding participants with missing TyG-BMI components may have introduced selection bias and limited generalizability, which multiple imputation of missing covariates could not address. Additionally, the potential violation of the proportional hazards assumption for household income in the healthy-to-FCMD group suggests that its association with FCMD risk may vary over time, warranting caution when interpreting its estimated hazard ratio as constant throughout follow-up. Finally, the complex CHARLS sampling design was not accounted for, potentially affecting variance estimation, and the use of separate Cox models precluded assessment of successive within-person disease-state transitions.

In summary, higher TyG-BMI was associated with a higher hazard of incident FCMD among participants without CMD at baseline and incident CMM among those with one CMD at baseline. These findings provide complementary observational evidence on cardiometabolic risk across different baseline CMD burdens. Future studies incorporating competing-risk and multistate approaches are warranted to further characterize these associations.

## Figures and Tables

**Table 1:** Baseline characteristics of the healthy-to-FCMD group and the FCMD-to-CMM group.

Characteristic	Healthy-to-FCMD Group (*n* = 5707)	FCMD-to-CMM Group (*n* = 2256)
TyG-BMI	196.7 (33.8)	212.6 (36.9)
Sex		
Female	3052 (53.5%)	1344 (59.6%)
Male	2655 (46.5%)	912 (40.4%)
Age (years)	58.3 (9.2)	61.2 (9.1)
Marital status		
Unmarried	647 (11.3%)	299 (13.3%)
Married	5060 (88.7%)	1957 (86.7%)
Residence		
Urban	1867 (32.7%)	821 (36.4%)
Rural	3840 (67.3%)	1435 (63.6%)
Education level		
Less than primary school	2733 (47.9%)	1115 (49.4%)
Primary school	1228 (21.5%)	518 (23.0%)
Middle school	1173 (20.6%)	432 (19.1%)
High school or above	573 (10.0%)	191 (8.5%)
Annual household income (yuan)	10,500.0 (2300.0, 31140.0)	10,600.0 (2300.0, 32400.0)
Ever drinking		
No	3432 (60.1%)	1389 (61.6%)
Yes	2275 (39.9%)	867 (38.4%)
Current drinking		
No	3670 (64.3%)	1603 (71.0%)
Yes	2037 (35.7%)	653 (29.0%)
Ever smoking		
No	3407 (59.7%)	1429 (63.3%)
Yes	2300 (40.3%)	827 (36.7%)
Current smoking		
No	3828 (67.1%)	1664 (73.8%)
Yes	1879 (32.9%)	592 (26.2%)
Sleep duration (hours)	6.4 (1.9)	6.3 (1.9)

Note: Continuous variables are expressed as mean (SD) or median (P25, P75); categorical variables as n (%). TyG-BMI: triglyceride-glucose-body mass index; FCMD: first cardiometabolic disease; CMM: cardiometabolic multimorbidity.

**Table 2:** Cox regression analysis of the association between TyG-BMI and outcome events in the two groups.

Group		Model 1		Model 2		Model 3	
		HR (95% CI)	*p*	HR (95% CI)	*p*	HR (95% CI)	*p*
Healthy-to-FCMD	TyG-BMI (per 1-unit increase)	1.007 (1.006, 1.009)	<0.001	1.009 (1.008, 1.010)	<0.001	1.010 (1.008, 1.011)	<0.001
	TyG-BMI (per 1-SD increase)	1.309 (1.261, 1.359)	<0.001	1.368 (1.316, 1.421)	<0.001	1.385 (1.332, 1.440)	<0.001
	Q1	1.000 (Ref.)		1.000 (Ref.)		1.000 (Ref.)	
	Q2	1.161 (1.031, 1.308)	0.014	1.227 (1.090, 1.383)	<0.001	1.236 (1.097, 1.393)	<0.001
	Q3	1.353 (1.204, 1.519)	<0.001	1.474 (1.310, 1.658)	<0.001	1.499 (1.332, 1.687)	<0.001
	Q4	1.967 (1.762, 2.197)	<0.001	2.198 (1.963, 2.462)	<0.001	2.268 (2.022, 2.544)	<0.001
	*p* for trend		<0.001		<0.001		<0.001
FCMD-to-CMM	TyG-BMI (per 1-unit increase)	1.007 (1.005, 1.008)	<0.001	1.007 (1.005, 1.009)	<0.001	1.007 (1.006, 1.009)	<0.001
	TyG-BMI (per 1-SD increase)	1.274 (1.197, 1.354)	<0.001	1.315 (1.234, 1.402)	<0.001	1.315 (1.232, 1.404)	<0.001
	Q1	1.000 (Ref.)		1.000 (Ref.)		1.000 (Ref.)	
	Q2	1.169 (0.970, 1.408)	0.101	1.219 (1.010, 1.470)	0.039	1.204 (0.997, 1.454)	0.054
	Q3	1.339 (1.116, 1.608)	0.002	1.384 (1.152, 1.663)	<0.001	1.372 (1.139, 1.653)	<0.001
	Q4	1.766 (1.481, 2.106)	<0.001	1.915 (1.597, 2.297)	<0.001	1.895 (1.574, 2.282)	<0.001
	*p* for trend		<0.001		<0.001		<0.001

Note: HR: hazard ratio; CI: confidence interval; SD: standard deviation; TyG-BMI: triglyceride glucose body mass index; FCMD: first cardiometabolic disease; CMM: cardiometabolic multimorbidity. Model 1: unadjusted; Model 2: adjusted for age and sex; Model 3: adjusted for age, sex, marital status, residence, education level, total annual household income, drinking status, smoking status, and sleep duration.

**Table 3:** Sensitivity analysis of the association between TyG-BMI and outcome events in the two groups.

Analysis	Healthy-to-FCMD				FCMD-to-CMM			
	N	Events	HR (95% CI)	*p*	N	Events	HR (95% CI)	*p*
Main analysis (fully adjusted)	5707	2599	1.010 (1.008, 1.011)	<0.001	2256	1040	1.007 (1.006, 1.009)	<0.001
Two-year landmark analysis	5138	2205	1.009 (1.008, 1.011)	<0.001	1996	875	1.007 (1.005, 1.009)	<0.001
Complete-case analysis	2898	1494	1.010 (1.008, 1.012)	<0.001	1176	601	1.006 (1.004, 1.008)	<0.001

Note: HR, hazard ratio; CI, confidence interval. Hazard ratios are presented per 1-unit increment of TyG-BMI. N and number of events for analyses based on multiple imputation were extracted from the first imputed dataset. In the two-year landmark analysis, participants with events or censoring within the first two years of follow-up were excluded, and time origin was reset at year 2. The complete-case analysis was performed without multiple imputation, restricted to participants with complete data for all covariates.

## Data Availability

The data can be accessed at https://charls.pku.edu.cn/ (accessed on 8 May 2025). Please follow the provided instructions to register as a user and obtain approval for data download. Once your request has been reviewed and approved, you will be able to access and download the data.

## Supplementary Materials

The following supporting information can be downloaded at https://www.mdpi.com/article/10.3390/healthcare14213722/s1, Table S1: Variable-specific missingness before multiple imputation in the two analytical groups; Table S2: Knot locations for restricted cubic spline analyses of TyG-BMI; Table S3: Associations between TyG-BMI and incident cardiometabolic multimorbidity according to baseline cardiometabolic disease type in the FCMD-to-CMM group.

## Abbreviations

The following abbreviations are used in this manuscript:

ARIC	Atherosclerosis Risk in Communities
BMI	body mass index
CHARLS	China Health and Retirement Longitudinal Study
CI	confidence interval
CMD	cardiometabolic disease
CMM	cardiometabolic multimorbidity
FCMD	first cardiometabolic disease
FPG	fasting plasma glucose
HbA1c	glycated hemoglobin A1c
HDL	high-density lipoprotein
HR	hazard ratio
IR	insulin resistance
LDL	low-density lipoprotein
MAPK	mitogen-activated protein kinase
MAR	missing-at-random
MICE	multiple imputation by chained equations
P25	25th percentile
P75	75th percentile
PI3-K/Akt	phosphatidylinositol 3-kinase/protein kinase B
Q1	first quartile
Q2	second quartile
Q3	third quartile
Q4	fourth quartile
RCS	restricted cubic splines
SD	standard deviation
TG	triglyceride
TyG	triglyceride-glucose index
TyG-BMI	triglyceride-glucose-body mass index
UK	United Kingdom
