# Supplementary material for: Association Between Triglyceride-Glucose-Body Mass Index and Cardiometabolic Multimorbidity Risk in Middle-Aged and Older Chinese Adults
Source: Healthcare (Basel). 2026 Nov 2;14(21):3722. doi: 10.3390/healthcare14213722 (PMC13763496; doi:10.3390/healthcare14213722)
Supplement: Supplementary file 1 [file healthcare-14-03722-s001.zip › healthcare-4604419-supplementary.pdf]

Supplementary Table S1. Variable-specific missingness before multiple imputation in the two analytical groups.

Variable	Healthy-to-FCMD (n = 5,707), missing n (%)	FCMD-to-CMM (n = 2,256), missing n (%)
Age	0 (0.00)	0 (0.00)
Sex	0 (0.00)	0 (0.00)
Marital status	57 (1.00)	27 (1.20)
Residence	0 (0.00)	0 (0.00)
Education level	103 (1.80)	47 (2.08)
Total annual household income	1,084 (18.99)	451 (19.99)
Ever drinking	74 (1.30)	34 (1.51)
Current drinking	69 (1.21)	31 (1.37)
Ever smoking	63 (1.10)	29 (1.29)
Current smoking	59 (1.03)	26 (1.15)
Sleep duration	274 (4.80)	117 (5.19)

Note:Data are presented as the number and percentage of missing observations [n (%)]. Missingness was assessed before multiple imputation, and percentages were calculated using the total number of participants in each analytical group as the denominator (n = 5,707 for the healthy-to-FCMD group and n = 2,256 for the FCMD-to-CMM group). Missing covariate values were subsequently handled using multiple imputation by chained equations (MICE), performed separately for the two analytical groups.

Supplementary Table S2. Knot locations for restricted cubic spline analyses of TyG-BMI.

Analytical group	Knot 1	Knot 2	Knot 3	Knot 4
Healthy-to-FCMD	148.43	179.65	206.54	260.23
FCMD-to-CMM	154.84	194.99	225.48	277.25

Note: Four knots were specified for TyG-BMI in each analytical group. Knot positions were determined separately based on the empirical distribution of TyG-BMI within each cohort.
FCMD, first cardiometabolic disease; CMM, cardiometabolic multimorbidity; TyG-BMI, triglyceride-glucose-body mass index.

Supplementary Table S3. Associations between TyG-BMI and incident cardiometabolic multimorbidity according to baseline cardiometabolic disease type in the FCMD-to-CMM group.

**Baseline CMD**	**Participants, n (%)**	**Incident CMM, n (%)**	**Adjusted HR (95% CI)**	**P value**	**P for interaction**	**Holm-adjusted P**
Hypertension	1,513 (67.07)	687 (45.41)	1.0065 (1.0043–1.0088)	<0.001	0.1784	0.5199
Diabetes	211 (9.35)	113 (53.55)	1.0050 (0.9992–1.0109)	0.091	0.3778	0.5199
Heart disease	464 (20.57)	211 (45.47)	1.0118 (1.0078–1.0158)	<0.001	0.1733	0.5199
Stroke	68 (3.01)	29 (42.65)	1.0268 (1.0105–1.0434)	0.003	0.0879	0.3515
Total	2,256 (100.00)	1,040 (46.10)	—	—	—	—

Overall interaction between TyG-BMI and the four-category baseline CMD variable: P = 0.0334.
Notes: Cohort B included participants with exactly one CMD at baseline. Incident CMM was defined as the first observed presence of at least two CMDs during follow-up.
Participants, n (%): percentages are calculated using all 2,256 participants in Cohort B. Incident CMM, n (%): percentages are calculated within each baseline disease subgroup.
Adjusted HRs (95% CIs) represent the association per 1-unit increase in TyG-BMI, estimated from separate fully adjusted Cox proportional hazards models within each baseline CMD subgroup. Models were adjusted for age, sex, marital status, residence, education, total annual household income, drinking status, smoking status, and sleep duration. Estimates were pooled across five multiply imputed datasets.
The overall interaction P value was obtained using the D1 pooled nested-model comparison for the TyG-BMI × four-category baseline CMD interaction (3 degrees of freedom). Disease-specific P for interaction values were obtained from separate exploratory comparisons of each baseline CMD category against the other three categories combined. Holm-adjusted P values account for these four exploratory tests.
Abbreviations: CMD, cardiometabolic disease; CMM, cardiometabolic multimorbidity; CI, confidence interval; HR, hazard ratio; TyG-BMI, triglyceride-glucose-body mass index.
